# RNA and DNA Binding Epitopes of the Cold Shock Protein *Tm*Csp from the Hyperthermophile *Thermotoga maritima*

**DOI:** 10.1007/s10930-020-09929-6

**Published:** 2020-10-22

**Authors:** Konstanze von König, Norman Kachel, Hans Robert Kalbitzer, Werner Kremer

**Affiliations:** Institut für Biophysik und Physikalische Biochemie, Universität Regensburg, 93040 Regensburg, Germany

**Keywords:** Cold-shock protein, *Tm*Csp, *Thermotoga maritima*, Single-stranded DNA, Single-stranded RNA, Protein/ssDNA complex, Protein/RNA complex, NMR spectroscopy, Temperature sensing

## Abstract

Prokaryotic cold shock proteins (CSPs) are considered to play an important role in the transcriptional and translational regulation of gene expression, possibly by acting as transcription anti-terminators and “RNA chaperones”. They bind with high affinity to single-stranded nucleic acids. Here we report the binding epitope of *Tm*Csp from *Thermotoga maritima* for both single-stranded DNA and RNA, using heteronuclear 2D NMR spectroscopy. At “physiological” growth temperatures of *Tm*Csp (≥ 343 K), all oligonucleotides studied have dissociation constants between 1.6 ((dT)_7_) and 25.2 ((dA)_7_) μM as determined by tryptophan fluorescence quenching. Reduction of the temperature to 303 K leads to a pronounced increase of affinity for thymidylate (dT)_7_ and uridylate (rU)_7_ heptamers with dissociation constants of 4.0 and 10.8 nM, respectively, whereas the weak binding of *Tm*Csp to cytidylate, adenylate, and guanylate heptamers (dC)_7_, (dA)_7_, and (dT)_7_ is almost unaffected by temperature. The change of affinities of *Tm*Csp for (dT)_7_ and (rU)_7_ by approximately 3 orders of magnitude shows that it represents a cold chock sensor that switches on the cold shock reaction of the cell. A temperature dependent conformational switch of the protein is required for this action. The binding epitope on *Tm*Csp for the ssDNA and RNA heptamers is very similar and comprises β-strands 1 and 2, the loop β1–β2 as well as the loops connecting β3 with β4 and β4 with β5. Besides the loop regions, surprisingly, mainly the RNA-binding motif RNP1 is involved in ssDNA and RNA binding, while only two amino acids, H28 and W29, of the postulated RNA-binding motif RNP2 interact with the uridylate and thymidylate homonucleotides, although a high affinity in the nanomolar range is achieved. This is in contrast to the binding properties of other CSPs or cold shock domains, where RNP1 as well as RNP2 are involved in binding. *Tm*Csp takes up a unique position since it is the only one which possesses a tryptophan residue instead of a usually highly conserved phenylalanine or tyrosine residue at the end of RNP2. NMR titrations suggest that neither (dT)_7_ nor (rU)_7_ represent the full binding motif and that non-optimal intercalation of W29 into these oligonucleotides blocks the access of the RNP2 site to the DNA or RNA. NMR-experiments with (dA)_7_ suggest an interaction of W29 with the adenine ring. Full binding seems to require at least one single purine base well-positioned within a thymine- or uracil-rich stretch of nucleic acids.

## Introduction

When microorganisms experience a sudden decrease in temperature, they suffer from a cold shock. Its most striking characteristic is the impairment of all growth–related processes within the cell. However, after an acclimatization period, a physiological cold shock response sets in, which allows for growth to be resumed and is therefore considered essential for the survival of unicellular organisms (for a review, see [1, 2]). This effect is based on the transient appearance of cold-induced proteins (CIPs). In particular, a subgroup comprising the so-called cold shock proteins (CSPs) is induced to an extreme extent.

CSPs are small, acidic proteins found in a wide variety of Gram-negative and Gram-positive species and are classified together as one family since they possess high sequence homology among each other (> 45%). They seem to exert their biological function by binding to single-stranded nucleic acids, especially RNA. In this way, secondary structures such as hair pins, which are increasingly formed in DNA and RNA at low temperatures, may be destabilized, thus enabling both transcription and translation to continue [3]. Due to their binding to mRNA with low sequence specificity, cold shock proteins are also called ‘RNA’ chaperones [3]. Because of their homology to the cold shock domains of the eukaryotic Y-box transcription factors, CSPs were originally thought to bind to the Y-box-sequence ATTGG. However, for *B. subtilis* CspB (*Bs*CspB) no apparent sequence specificity was found [4, 5]. More recent experiments have revealed that *Bs*CspB possesses a strong preference for polypyrimidine ssDNA [5]. Its affinity is highest for thymidylate-rich stretches of DNA and directly correlates with the number of T-bases present [6]. In this respect, (dT)_7_ is sufficient for obtaining nanomolar dissociation constants [7].

The tertiary structures of a number of cold shock proteins have been solved, i.e., of Csp from hyperthermophilic *Thermotoga maritima* [8], of thermophilic *Bacillus caldolyticus* [9], or of CspB from mesophilic *Bacillus subtilis* [10, 11], and of CspA from mesophilic *Escherichia coli* [12, 13]. They belong to the OB (oligonucleotide/oligosaccharide binding) fold family, which typically is found in Greek-key β-barrel proteins. In their primary structures, CSPs contain the sequence motifs RNP1 and RNP2 commonly found in RNA-binding proteins [14, 15]. Taken together, these structural features further support their role as nucleic acid binding proteins.

The crystal structures of CspB from the mesophilic Bacillus subtilis (*Bs*CspB) complexed with the hexanucleotide 5′-UUUUUU-3′ and the pentanucleotide 5′-GUCUUUA-3′ single stranded RNA (ssRNA) were published recently [16]. Here, one CSP is complexed with one ssRNA-molecule. In contrast, the ssDNA 5′-TTTTTT-3′ (dT)_6_ is bridging to two *Bs*CspB molecules in the crystal structure [17]. A solution NMR structure of *Bs*Csp in complex with the ssDNA dT_7_ shows that in solution the binding mode of ssDNA is probably different to that observed in X-ray crystallography [18]. Crystal structures of CSP-nucleotide complexes of hyperthermophilic bacteria have not yet been published.

In this paper we aim to characterize the nucleic acid interactions of the cold shock protein from the hyperthermophilic bacterium *T. maritima* (*Tm*Csp) with both ssRNA and ssDNA oligonucleotides. A thorough analysis was considered necessary because it had been shown that the binding specificity among the different CSPs varies slightly [19]. Cold chock proteins have two main functions after cold shock application, (1) an inhibition of the bulk protein expression and (2) an increased expression of CSPs themselves and other cold shock induced, specific proteins (for a review see e.g. [2]). At the millimolar concentrations of CSPs obtained at cold shock conditions, transcription as well as translation is downregulated completely as studies in cell free systems show [20]. For *Tm*Csp 50% inhibition of the two processes is obtained at a concentration of 140 μM. Inhibition can completely be abolished by addition of hepta-2′-desoxy-thymidylate (dT)_7_ that competitively binds to *Tm*Csp with high affinity [20]. Specific activation of translation by high affinity binding of *Ec*CspA to the 5′-untranslated region of the CspA mRNA can be observed in cell-free systems at cold shock temperatures largely independent of the coding sequence used [21]. For *Tm*Csp detailed data about the unspecific bulk effects and the sequence specific activation effects are still missing, especially with regard to the question if binding to RNA or DNA involves different amino acid motifs of the protein. Preliminary studies had indicated that *Tm*Csp would be an ideal candidate for such studies since its binding affinity for DNA seemed to be significantly higher than those of other CSPs. Furthermore, it could be deduced that *Tm*Csp appeared to require oligomers comprising seven nucleotides as a minimum binding site.

According to the findings that ssDNA binding by *Bs*Csp predominantly depends on the base composition of the template and not on the specific base sequence [5], simplified oligonucleotide sequences were used for the experiments presented in the following, which consisted of only one type of nucleotide each.

## Results

### Nucleotide Binding Properties of TmCsp Monitored by Electrophoretic Mobility Shift Assay (EMSA)

**Fig. 1:**
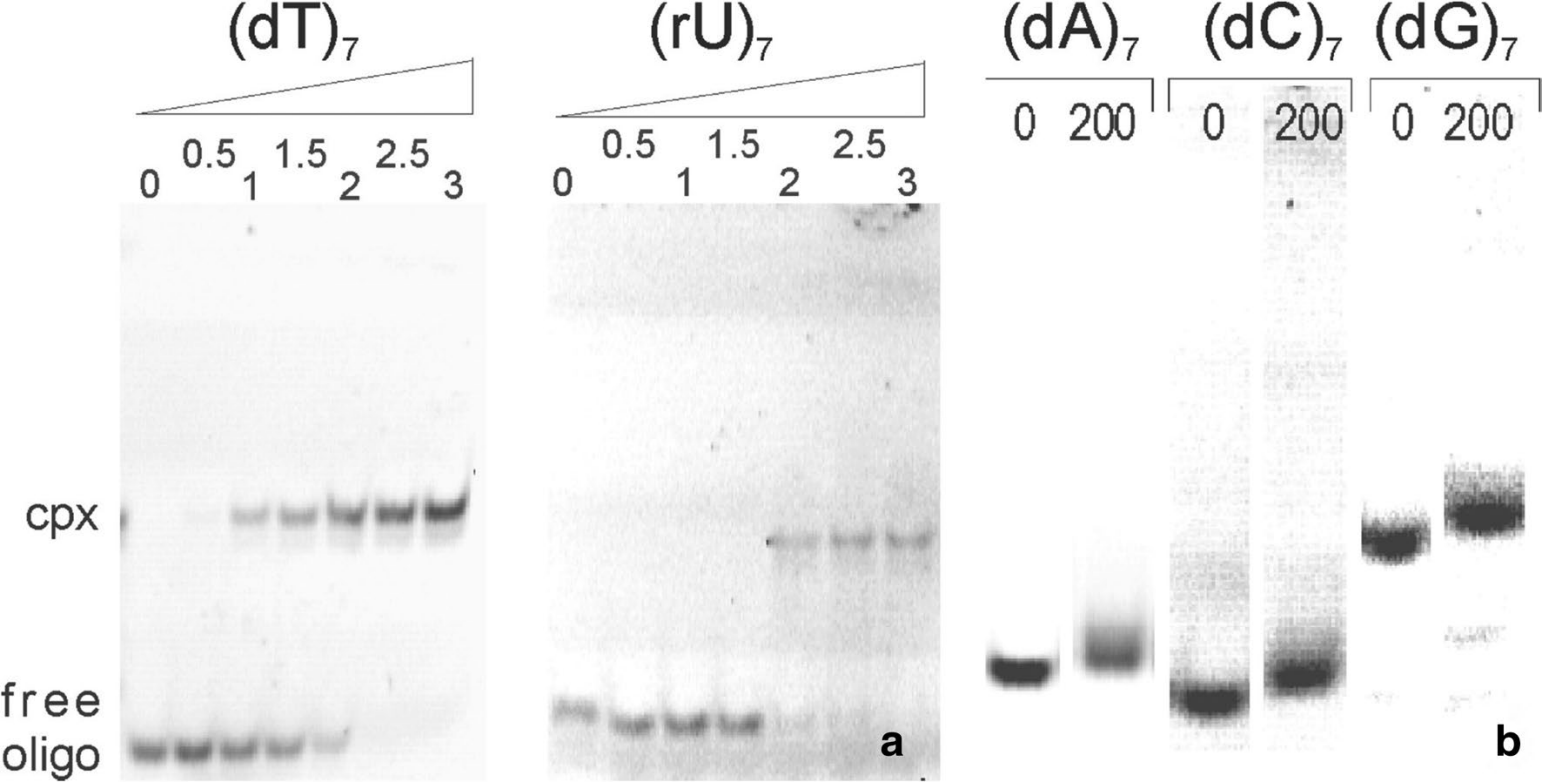
Relative binding affinities as observed by electrophoretic mobility shift assays. **a** Gels run after the incubation of 0.5 μM (dT)_7_ or (rU)_7_ with *Tm*Csp in different concentrations at room temperature. The molar ratio of protein : oligonucleotide is indicated above the gel. In both cases, good binding interactions were observed. Cpx: Complex. **b** Incubation of 0.5 μM (dA)_7_, (dC)_7_ or (dG)_7_ without *Tm*Csp and with *Tm*Csp at a ratio of DNA:protein = 1:200. Note that the vertical scale was increased by a factor of 2 for visualizing smaller shift changes with addition of Csp. No band could be observed characteristic for complex formation

To investigate which types of nucleotides are recognized as *Tm*Csp substrates, binding studies were performed with a non-radioactive electrophoretic mobility shift assay. Constant amounts of 5′-fluorescein-labeled oligonucleotides were incubated with varying concentrations of *Tm*Csp. In the case of the pyrimidine oligonucleotides (dT)_7_ or (rU)_7_, increasing concentrations of *Tm*Csp resulted in a gradual decrease of the intensity of the band corresponding to the free oligonucleotide, while an additional double band reciprocally appeared in the gel with a smaller electrophoretic mobility (Fig. 1a). Silver-staining of the gels showed that both bands contain protein. From this it was concluded that the shifted bands at high oligonucleotide concentrations correspond to the nucleoprotein complex (data not shown). Complete retardation of the free oligonucleotides occurred at a molar ratio of *Tm*Csp: (dT)_7_ or (rU)_7_ of 2:1–2.5:1. In contrast, incubation of the cold shock protein with the pyrimidine oligonucleotide (dC)_7_ as well as the purine oligonucleotides (dA)_7_ or (dG)_7_ at a ratio as high as 200:1 (corresponding to 100 μM *Tm*Csp) did not lead to a band shift characteristic for a stable nucleoprotein complex at the concentrations of oligonucleotides (0.5 μM) used in this assays (Fig. 1b). The purine oligonucleotides were re-tested at 300 μM of *Tm*Csp but no band shift at lower migration height was observed that contained protein as determined by Silver staining (data not shown).

### One Dimensional NMR Studies of the ssDNA Binding Interactions

**Fig. 2:**
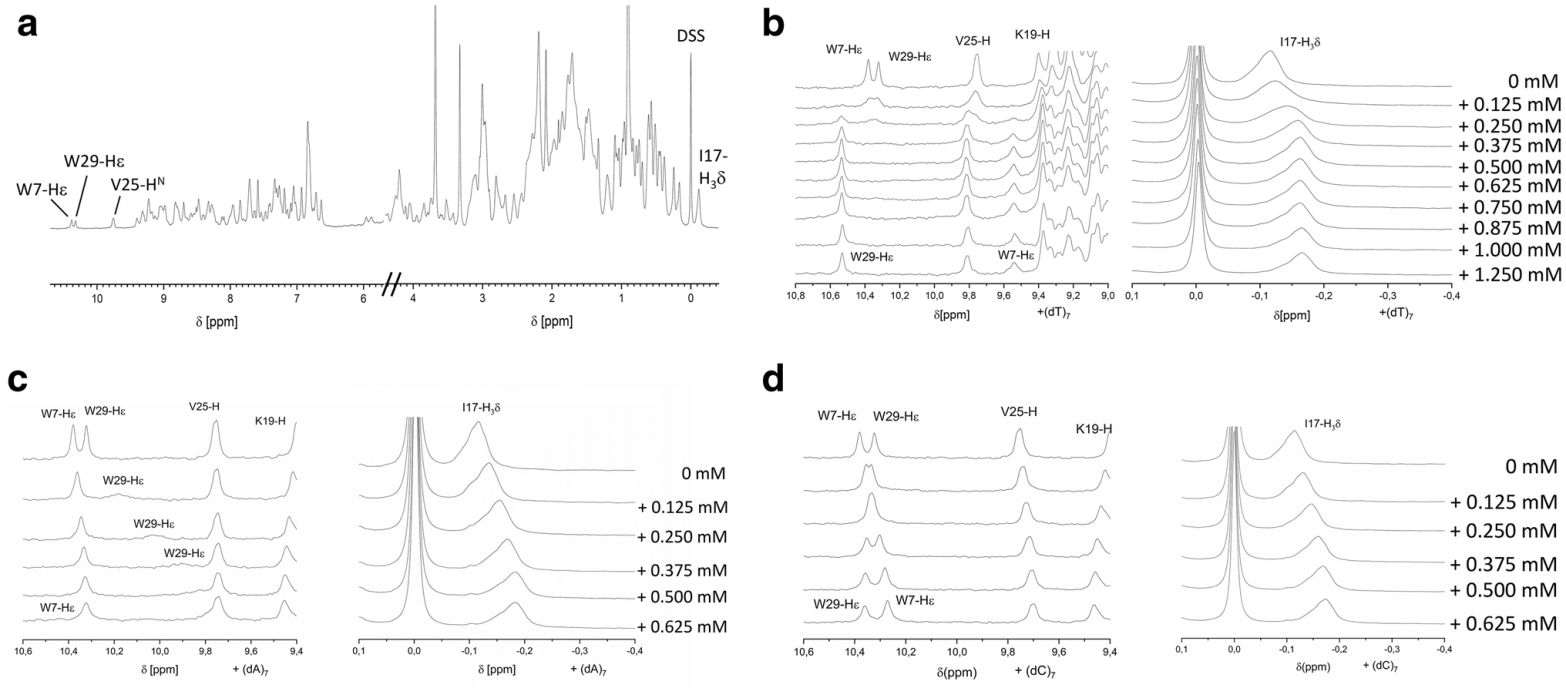
1D ^1^H NMR spectra of *Tm*Csp in the absence and presence of ssDNAs. **a** Overview spectrum of 0.5 mM *Tm*Csp in 50 mM sodium phosphate, pH 6.5, 20 mM NaCl, 0.2 mM EDTA, and 0.1 mM DSS at 303 K. **b** to **d** 1 mM *Tm*Csp in 50 mM sodium phosphate, pH 6.5, 20 mM NaCl, 0.2 mM EDTA, 0.1 mM DSS, and varying nucleotide concentrations as indicated in the plots. Temperature 303 K. **b** Spectral changes induced by the addition of (dT)_7_, **c** of (dA)_7_, and **d** of (dC)_7_ in varying concentrations as indicated

Moreover, binding interactions were analyzed by NMR spectroscopy to define the regions of the protein involved in complex formation. An aqueous solution of *Tm*Csp (0.5 mM) in sodium phosphate buffer was separately titrated with (dA)_7_, (dC)_7,_ or (dT)_7_ in the range of 0 to 0.625 mM by adding equal amounts of oligonucleotide in each step and recording a 1D ^1^H NMR spectrum of each of these samples at 303 K. We initially intended to use the oligonucleotides (dA)_7_ and (dC)_7_ merely as controls since they do not strongly interact with *Tm*Csp in the shift assay (see above). However, one has to note that in the NMR-experiments the protein and nucleotide concentrations are higher by almost three orders of magnitude compared to the shift assays. It can be expected that under these conditions relatively weak interactions are also visible as, indeed, we will demonstrate in the following. Without DNA, the protein spectrum was well resolved and displayed sharp signal lines (Fig. 2a). Depending on the type of oligonucleotide added, the protein underwent distinct changes in each titration series, which was clearly reflected in the 1D spectra.

In the presence of (dT)_7_, the resonances broadened all over the spectrum (including also the signals of non-exchangeable protons). The resonance lines of the ε1-side chain protons of the tryptophan residues W7 and W29, of the main chain amide proton of V25, and of the high-field shifted H^δ^ of I17 (Fig. 2b) at 10.45, 10.35, 9.77, and − 0.146 ppm, respectively, are of special interest since they encountered significant changes upon nucleotide binding which could be easily monitored due to their isolated position. In the first titration step, these signals broadened severely (Fig. 2b). Increased (dT)_7_ concentrations first led to a splitting of the signal of V25 and I17; at high nucleotide concentrations only one downfield shifted signal remains as it is typical for a slow exchange process. A similar process is observed for the tryptophan side chain resonances. At increasing (dT)_7_-concentrations a new downfield shifted resonance at 9.457 ppm and a high field shifted resonance at 10.536 ppm appear and the intensity of the lines corresponding to nucleotide-free Csp simultaneously get weaker. The chemical shifts of these signals are virtually independent of the oligonucleotide concentration as to be expected for slow exchange processes. In slow exchange, the assignment of new resonances is not straightforward without additional information. When during a titration a new peak in close vicinity of the original peak is created and the total integral of the two peaks is constant, one can assume that they correspond to the same residue in the free and complexed form. With this argument, the downfield shifted signal at 9.81 ppm and the high-field shifted resonance at − 0.17 ppm, respectively, can be assigned to the amide proton of V25 and the methyl protons of I17 in the complex. In the *Bs*CspB-dT_7_ complex the side chain resonance of W8 (corresponding to W7 in *Tm*Csp) is shifted upfield by approximately 1 ppm. By analogy, it is likely that the signal at 9.457 ppm corresponds to W7 in the *Tm*Csp nucleotide complex and the signal at 10.536 ppm to W29.

Above a molar ratio of approximately 1:2 of (dT)_7_:*Tm*Csp, the signals of free *Tm*Csp are completely abolished and the integrals corresponding to complexed protein do not change anymore. Consistently, even at the endpoint of the titration with a (dT)_7_ concentration of 1.25 mM, the resonance line of W29 is broadened by a factor of 1.25 compared to the oligonucleotide free sample. Note that doubling the molecular mass by dimerization would lead to an increase of the rotational correlation time and thus of the approximate line width by a factor of 1.26.

Titration of *Tm*Csp with (dA)_7_ leads again to significant spectral changes proving that at the high concentrations used for NMR the oligonucleotide interacts with the protein. When again considering the 4 resonances that were used for monitoring the binding of (dT)_7_, spectral changes are also observed but now typical for fast exchange processes (Fig. 2c). Addition of (dA)_7_ leads to a very small continuous upfield shift of the resonance line of W7 accompanied by a significant line broadening by a factor of 1.8. Similar effects are observed for V25 and Ile17. Both of them are upfield shifted with increasing nucleotide concentration to positions close to those observed for TmCsp ligated with (dT)_7._ In contrast to the titration with (dT)_7_, the signal of W29 is continuously shifted upfield and severely broadens. At a ratio of approximately 1:0.375 of *Tm*Csp:(dA)_7_, it is broadened almost beyond detection. In fact, under fast to moderately fast exchange conditions a stronger exchange broadening effect is to be expected for lines with a larger chemical shift difference in the two states involved (nucleotide-free and nucleotide-bound). At oligonucleotide concentrations higher than 0.5 mM a saturation effect is observed for all considered resonances. However, the signal of W29 could not be identified at saturation conditions suggesting an additional heterogeneity of the interaction mode at high oligonucleotide concentrations.

As in case of (dA)_7_ spectra, continuous chemical shift changes are observed when the oligonucleotide (dC)_7_ was added, indicating again a protein-nucleotide interaction, fast on the NMR time scale (Fig. 2d). As with (dA)_7_ W7, V25, and I17 are shifting downfield. However, W29 is downfield shifted as observed in the case of (dT)_7_. No larger exchange broadening is observed, but at the highest DNA concentration used (0.625 mM), the line widths are increased by a factor of 1.3. Again a saturation behavior is observed for all of these amino acids. (dG)_7_ did not yield well-resolved spectra and was therefore omitted from analysis.

In conclusion, these spectral changes indicate interactions of the cold shock protein with all ssDNA fragments studied. It is clear that the extent of the line broadening and the changes in chemical shift of the protein resonances upon binding are different. The main difference between the gel shift assays and the NMR spectra shown here are the concentrations used. At the high concentrations used in NMR (1 mM protein and ≥ 0.625 mM nucleotide) and the lack of shear force as exerted in EMSA by the electric field, low-affinity interactions with all oligonucleotides were detectable which could not be detected by the electrophoretic mobility shift assay shown above with oligonucleotide concentrations of 0.5 μM. Surprisingly, a single oligonucleotide molecule binds two Csp-molecules.

### Identification of (dT)_7_ and (rU)_7_ Binding Epitopes by Two-Dimensional NMR

**Fig. 3:**
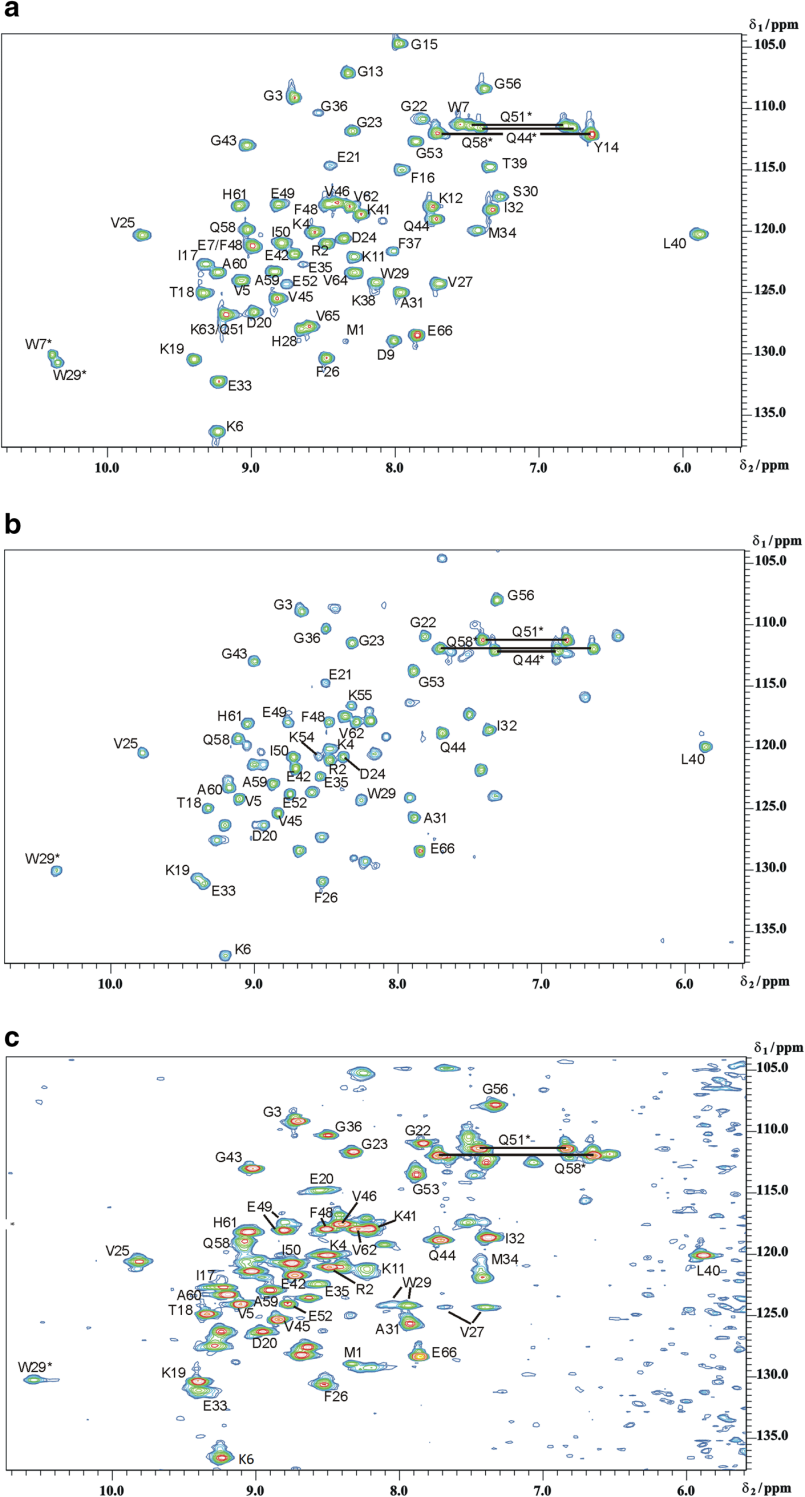
Changes in the [^1^H-^15^N]-HSQC spectrum of ^15^N-*Tm*Csp induced by the oligonucleotides (dT)_7_ and (rU)_7_. The samples contained 1 mM uniformly ^15^N-enriched *Tm*Csp in 50 mM sodium phosphate, pH 6.5, 20 mM NaCl, 0.2 mM EDTA, and 0.1 mM DSS, temperature 303 K. The last step of the titration with the RNA (upper spectrum) and the DNA (lower spectrum) is shown at a ratio of *Tm*Csp:oligonucleotide = 1:0.625. (top) *Tm*Csp only, (middle) *Tm*Csp with (rU)_7_, (bottom) *Tm*Csp with (dT)_7_. (*), side chain resonances

The complex formation was monitored on a residue-by-residue basis by recording a series of ^1^H–^15^N HSQC fingerprint spectra of ^15^N-enriched *Tm*Csp samples in the presence of increasing amounts of the oligonucleotides (dT)_7_ or (rU)_7_ at 303 K. Resonance assignments of free *Tm*Csp were performed on the basis of the assignments published by Kremer et al. [8]. The spectral changes due to the addition of oligonucleotide were tracked by comparison of the successively recorded [^1^H, ^15^N] HSQC spectra (Fig. 3).

**Fig. 4:**
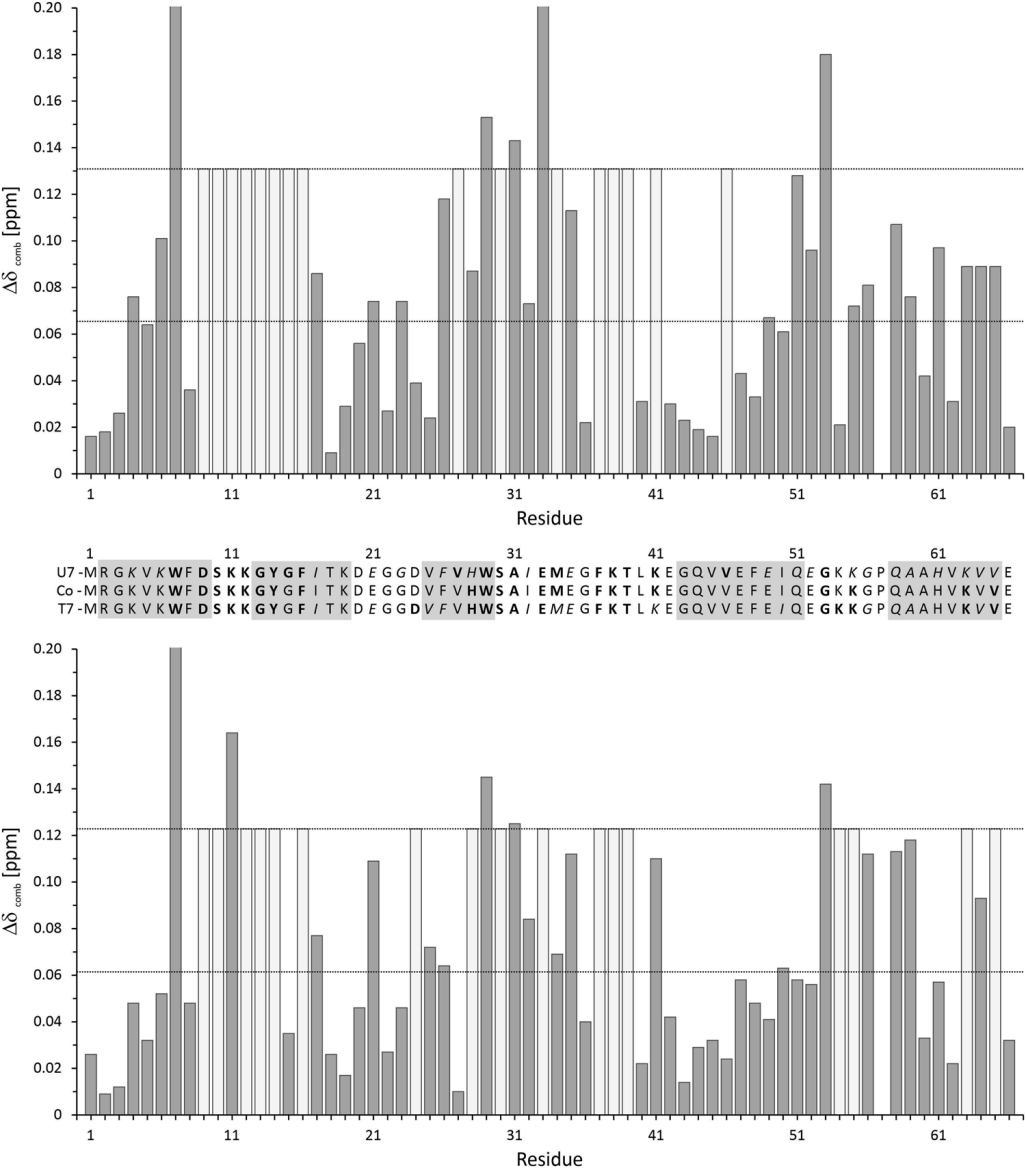
Residue specific chemical shift perturbations by the oligonucleotides (dT)_7_ and (rU)_7_. The residue and amino acid specific combined chemical shift perturbations ∆δ_comb_ [37] for the (rU)_7_ RNA (top) and (dT)_7_ ssDNA (bottom) were calculated from the 1D and 2D spectra in the absence of oligonucleotides and in the presence at molar ratios of *Tm*Csp:oligonucleotide = 1:0.625 (dark grey bars). The horizontal lines depict standard deviations σ_0_ and 2 σ_0_. The residues whose unperturbed resonances disappeared in the presence of oligonucleotides and could not be identified in the spectra are presented as light grey bars. Their value was set to 2 σ_0_. The missing entry in position 57 represents a proline residue (no amide proton). (Middle) In addition, residues are marked as interaction partners in the *Tm*Csp sequence with moderate probability (cursive letters, σ_0_ ≥ ∆δ_comb_ < 2 σ_0_) and with high probability (bold letters, ∆δ_comb_ ≥ 2 σ_0_). (Co) indicates residues that have high interaction probability for at least one oligonucleotide and moderate to high probability for the second nucleotide. Boxes indicate the limits of β-strands in *Tm*Csp

**Fig. 5:**
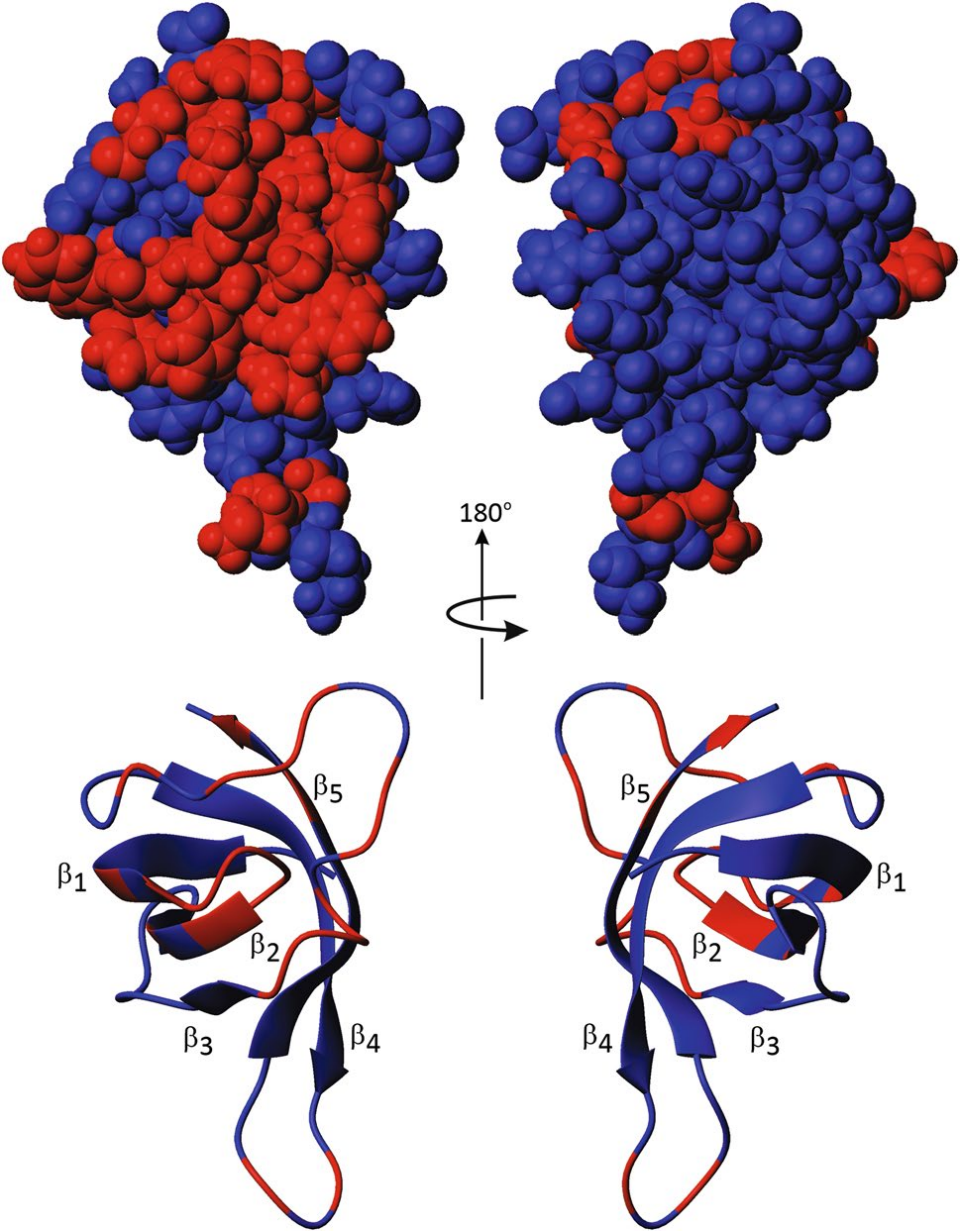
Mapping of the chemical shift perturbations caused by oligonucleotide binding. Residues that probably are involved (red) or not involved (blue) by oligonucleotide binding (see Fig. 4) were mapped onto the 3D model of pure *Tm*Csp [8]. These representations were generated using the program *MOLMOL* [35]

Upon complex formation with (dT)_7_, the backbone and side chain amide resonances of the protein in the [^1^H,^15^N] HSQC spectra shifted to different extents. In addition, many peaks became undetectable. Such an effect was observed for the amide resonances of W7, D9, S10, K12, G13, Y14, F16, D24, H28, S30, E33, F37, K38, T39, K41, Q51, K54, K55, K63, and V65 (Fig. 4). It could be the effect of strong line broadening by intermediate exchange or it could correspond to the slow exchange effects observed for the H^ε1^ of W7 where the signal in the bound state shifts considerably in the 1D-spectra but cannot be identified unequivocally in the 2D-spectra. The combined chemical shift changes ∆δ_comb_ including the backbone amide nitrogen and proton resonances as well as some side chain resonances are depicted in Fig. 4. Amino acids whose resonances shifted after the interaction with the oligonucleotides (dT)_7_ by more than σ_0_ and 2 σ_0_ were considered moderately and very probable interacting sites, respectively (Fig. 4). The [^1^H,^15^N]-HSQC spectrum of the complex with (rU)_7_, is much better defined than that with (dT)_7_. As already observed for (dT)_7_ upon complex formation with (rU)_7_, the backbone and side chain amide resonances of the protein in the [^1^H,^15^N] HSQC spectra shifted to different extents. As in the case of (dT)_7_ many peaks became undetectable or shifted considerably. During the titration with (rU)_7_ also significant chemical shift changes ∆δ_comb_ were observed. In most cases they were associated with the same residues as already described for the interaction with (dT)_7_ (Fig. 4). A consensus interacting surface was derived by using only residues that are most likely interacting in at least one of the complexes and in the other structure have at least an intermediate interaction probability (Fig. 4). They are labeled in the consensus plot Co in Fig. 4 in bold letters. These residues are plotted on the NMR structure of *Tm*Csp determined at 303 K (Fig. 5).

**Fig. 6:**
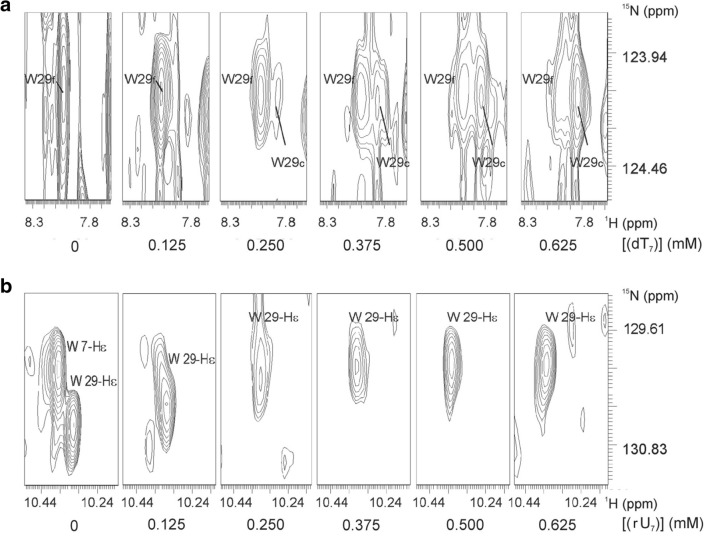
Exchange time scales in the complexes *Tm*Csp-(dT)_7_ and *Tm*Csp-(rU)_7_. 1 mM uniformly ^15^N-enriched *Tm*Csp in 50 mM sodium phosphate, pH 6.5, 20 mM NaCl, 0.2 mM EDTA, 0.1 mM DSS, and varying nucleotide concentrations. **a** Slow exchange of the side chain amide resonance of W29 in the complex with (dT)_7_: By the addition of (dT)_7_, the signal of the main chain amide proton of W29 in free *Tm*Csp (W29_f_) disappears, whereas the signal of W29 in complexed *Tm*Csp (W29_c_) appears simultaneously. **b** Fast exchange of the main chain amide resonance of W29 in the complex with (rU)_7_: Interaction with (rU)_7_ leads to a continuous shift of the signals as seen e.g. for the side chain amide proton of W29

In the complex of Csp with (rU)_7_ many resonances remained either unaffected or shifted continuously with increasing oligonucleotide concentration, indicating that fast or moderately fast exchange conditions prevailed. However, in the presence of (dT)_7_, the chemical exchange rates of many of these resonances of *Tm*Csp behaved in a way which is typical for intermediate exchange or slow exchange on the NMR time scale (Figs. 2, 3, 4). This result reflects most probably that a smaller exchange correlation time of the complex leads to slow exchange conditions. It is also supported by the fact that the overall dissociation constant of *Tm*Csp for (dT)_7_ is 2.7-fold lower than the one for (rU)_7_. An example for slow exchange is shown in Fig. 6a, where the backbone H^N^-resonance signal of W29 in free *Tm*Csp becomes weaker and broader while simultaneously a new resonance signal of W29 in complex with the oligonucleotide appears and becomes stronger.

In contrast, many resonances in the complex *Tm*Csp-(rU)_7_ show typical fast exchange behavior with continuously shifting cross peaks during the titration, probably since the exchange correlation time is smaller. This is illustrated in Fig. 6b for W29 again, where the H^ε1^-resonance of W29 shifts with increasing oligonucleotide concentrations. The H^ε1^-resonance of W7 is strongly broadened at a concentration of 0.125 mM (rU)_7_ already and then disappears completely.

### Determination of the Dissociation Constants of the *Tm*Csp-RNA/ssDNA-Complexes by Trp Fluorescence Quenching

The *K*_D_ values for the binding of (dT)_7_ and (rU)_7_ by *Tm*Csp were determined by fluorescence quenching titration experiments. Tryptophan residues contribute most to the fluorescence emission spectrum and were used to monitor the binding process. In order to determine the affinities, the protein fluorescence intensity was measured as a function of the oligonucleotide concentration at three different temperatures. The dissociation constants were then calculated from the experimental binding isotherms using a stoichiometry of 2:1. This stoichiometry initially derived from the NMR-data has been confirmed by the fluorescence quenching titration experiments. Titration curves were all monophasic, indicating that the ligands ((dA)_7_, (dC)_7_, (dG)_7_, (dT)_7_ or (rU)_7_) bound to the two Csp molecules with equal affinity. Hence identical binding sites were assumed in the fit model (Table 1).In line with that, the ligand binding of either (dA)_7_, (dC)_7_, (dG)_7_, (dT)_7_ or (rU)_7_, does not show any protein NMR peaks characteristic for non-equivalent positions in the two Csp molecules in the complex wit the oligonucleotide. Strikingly, the *K*_D_ values for the oligonucleotides shown to be strongly bound in the gel shift assays ((dT)_7_ and (rU)_7_)) were in the nanomolar range at 303 K. For all other oligonucleotides the *K*_D_ values lay in the micromolar range (Table 1). In addition, the effect of temperature upon binding is very different for different oligonucleotides. At 30 °C, the affinities of the protein for (dT)_7_ were approximately 400 fold higher than at 70 °C, and about 1700 fold higher for (rU)_7_, which is in excellent agreement with the idea that one physiological function of *Tm*Csp is temperature sensing. For (dA)_7_, (dC)_7_ and (dG)_7_, the affinities increased only about 1.5–5 fold when the temperature was reduced.

**Table 1:** Dissociation constants of *Tm*Csp complexed with various nucleotide heptamers at different temperatures

Oligonucleotide	*K*_D_ (303 K) [µM]	*K*_D_ (323 K) [µM]	*K*_D_ (343 K) [µM]
(dA)_7_	5.0 ± 0.2	19.2 ± 0.5	25.2 ± 0.6
(dC)_7_	2.8 ± 0.1	n.d.	11.0 ± 0.1
(dG)_7_	3.4 ± 0.1	n.d.	5.6 ± 0.1
(dT)_7_	(4.0 ± 0.2)·10^−3^	0.44 ± 0.02	1.6 ± 0.04
(rU)_7_	(10.8 ± 0.8)·10^−3^	1.52 ± 0.01	17.4 ± 0.1

The dissociation constants were determined by quenching of the tryptophan fluorescence (see Materials and Methods). For data evaluation, two binding sites for *Tm*Csp per heptamer were assumed. *K*_D_ is the dissociation constant for one binding site assuming independent binding. 343 K (70 °C): physiological temperature of *T. maritima*. 323 K (50 °C): temperature range in which the cold shock sets in. 303 K (30 °C): cold shock. *n.d.* not determined

### Stoichiometry of Binding Derived from NMR Spectroscopy

**Fig. 7:**
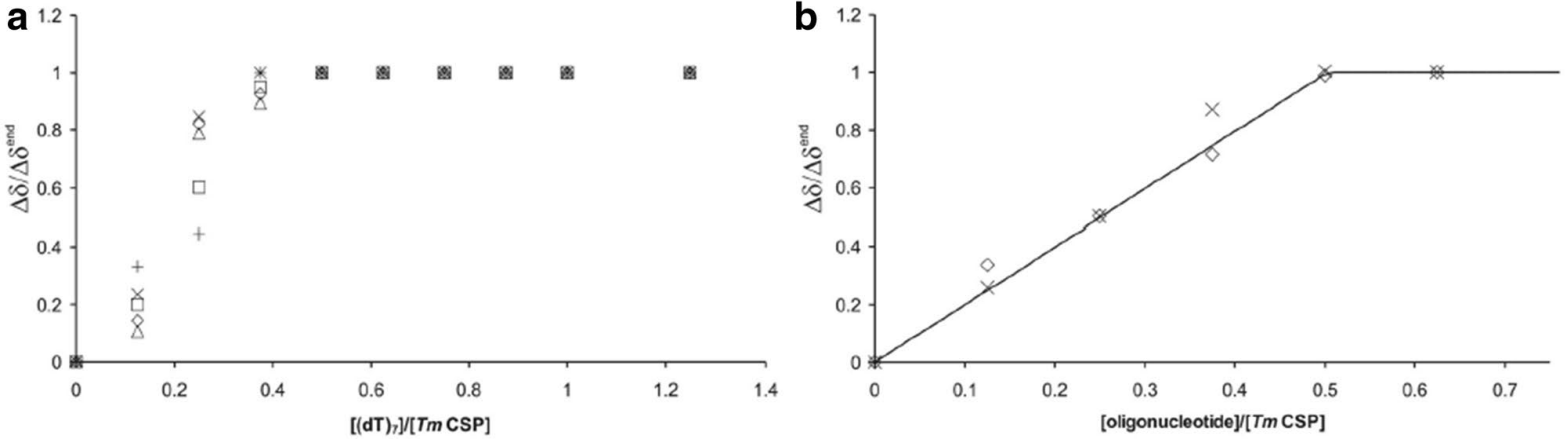
Stoichiometries of nucleic acid binding from NMR data. **a** Relative chemical shift change for isolated protein signals in 1D ^1^H NMR spectra caused by addition of (dT)_7_ (see Fig. 2) indicating a stoichiometry of two *Tm*Csp molecules per (dT)_7_. The chemical shifts were normalized by setting the chemical shift at the highest (dT)_7_.concentration to 1. **b** Mean values of the relative chemical shift change taken from HSQC titration experiments (see Fig. 3) with (dT)_7_ (◊) and (rU)_7_ (X). The solid line symbolizes the theoretical binding curve as defined by (Eqs. 2 and 4) with *N* *=* 2 and Δ*δ/*Δ*δ*^end^ = 1

In principle, NMR spectroscopy allows the quantification of oligonucleotide-protein interactions and the evaluation of different binding models, e.g., cooperative binding or independent binding to several binding sites can be distinguished. Since the *K*_D_ values for (dT)_7_ and (rU)_7_ binding as determined by the fluorescence quenching studies are in the nanomolar range and the protein and oligonucleotide concentrations used in the NMR experiments were in the millimolar range, the *K*_D_ could be neglected for the quantitative analysis of the chemical shift changes observed during the oligonucleotide titrations (see Eqs. 2, 3 and 4 in Materials and Methods). Figure 7a shows the normalized chemical shift changes for isolated protein signals in 1D ^1^H NMR spectra taken from a (dT)_7_ titration experiment with a molar ratio of (dT)_7_:*Tm*Csp up to 1.25:1. The data were fitted by using the *K*_D_-values obtained from fluorescence quenching. The saturation effect of the chemical shift change at a ratio of 0.5 indicates a stoichiometry of two molecules *Tm*Csp per (dT)_7_. These chemical shift changes are in perfect agreement with the intensity changes of residues involved in slow exchange described above. We further averaged the normalized ^1^H and ^15^N chemical shift changes of clearly observable resonances in fast exchange obtained from the HSQC titration experiments. The data were fitted using the *K*_D_-values from fluorescence quenching for (dT)_7_ and (rU)_7_ and eqs. 2 and 3. We got values of 1.99 ± 0.05 ((dT)_7_) and 2.21 ± 0.04 ((rU)_7_), respectively, for the number of independent binding sites *N*. The average maximal chemical shift changes *Δδ*^*end*^, led to *Δδ/Δδ*^*end*^ of 0.93±0.12 ((dT)_7_) and 0.94±0.10 ((rU)_7_), respectively (see Fig. 7a and b). So, the stoichiometry is close to two molecules *Tm*Csp per heptanucleotide.

## Discussion

### Stoichiometry of Oligonucleotide Binding and Size of the Oligonucleotide Binding Site

The titration experiments consistently prove that under cold shock conditions (303 K) two molecules of *Tm*Csp are bound per uridylate or thymidylate heptamer (Figs. 1, 2 and 7). The same also holds for (dA)_7_ and (dC)_7_ (Fig. 2).The concentrations of Csp and the oligonucleotides were carefully checked (see Materials and Methods) and could be confirmed directly by NMR spectroscopy.

No hints for a stronger interaction between the two Csp molecules are visible in the NMR spectra. We conclude that their mutual interaction must be very weak or that it must have an internal symmetry leading to equivalent chemical shifts of a given nucleus in the two proteins. As a consequence the length of the oligonucleotide binding site is only 3 nucleotides per Csp (or, alternatively, for a dimer binding to 6 of the 7 nucleotides). In fact, dimerization in the absence of oligonucleotides was observed for *Bs*CspB in crystals [13] and in solution [22] via strand β4, for *St*CspE from *Salmonella typhimurium* [23]. Most interesting is the interaction of a mutant of *Bc*CspB from *Bacillus caldolyticus* [24] through its nucleic acid-binding surface in a point symmetrical arrangement. The latter structures form a symmetric dimer with antiparallel orientation that would lead to the same chemical shifts of nuclei in equivalent positions in the two Csps in the dimer.

When compared with the binding properties of the cold shock proteins from other species, these findings let us suggest that Csp from the hyperthermophilic organism *T. maritima* takes up a unique position because of its unusual origin. It represents a Csp homologue, which previous results cannot be transferred to. For example, chemical shift mapping with CspA from *E. coli* [12] and CspB from *B. subtilis* [18] demonstrated that both the RNP1 and the RNP2 motifs strongly contribute to DNA binding. Analysis of the binding stoichiometries showed that the binding sites for *Ec*CspA and *Bs*CspB on homogenous templates consist of 6–7 nucleotides per Csp molecule [5, 16].

### Identification of Interaction Sites

Chemical shift mapping as well as mapping of intensity changes due to slow and intermediate exchange effects show that the location of the binding epitopes for the pyrimidine based ssDNA or RNA oligomers (dT)_7_ and (rU)_7_ on *Tm*Csp do not differ significantly (Figs. 4 and 5). The epitope mainly comprises the last amino acids of β-strand 1 (W7, D9) and the first amino acids of β-strand 2 (G13, Y14, F16) and the loop region in between the two strands (S10, K11, K12). Further interaction points include H28 and W29, the last amino acids of strand β3, and are scattered along the long loop connecting β3 and β4 (S30, A31, E33, M34, F37, K38, T39, K41), and the loop between β4 and β5 (G53, K55). In strand β5 K63 and V65 see the oligonucleotide binding. Surprisingly, these binding sites only partly involve the classical RNA-binding sites, which had been predicted from the sequence alignment of 23 cold shock domains known from other CSPs and which consists of the motifs RNP1 (residues 13–20) and RNP2 (residues 25–29) [4, 12, 25, 26]. In RNP1 G13, Y14, and F16 are concerned, in RNP2 only the aromatic amino acids H28 and W29. Nevertheless, a high binding affinity in the nanomolar range is achieved at cold shock conditions.

Judging from the *K*_D_ values and the gel shift assays, the other oligonucleotides interact significantly weaker with *Tm*Csp. Nevertheless at least for the two oligonucleotides (dA)_7_ and (dC)_7_ interactions with *Tm*Csp can be detected at the high concentrations used in the 1D NMR experiments. A detailed analysis of their binding by 2D-NMR spectroscopy has not been performed. Concerning the side chains of the two tryptophan residues qualitatively the same behavior is observed, both nucleotides influence the shifts of the H^ε1^-resonance of W7 and W29, but size and direction of the shifts vary considerably. A moderate downfield shift of the H^ε1^-resonance of W29 is induced by (dT)_7_ and a small upfield shift by (dC)_7_ but a large upfield shift is caused by binding of (dA)_7_ (Fig. 2). For all three nucleotides upfield shifts of the H^***ε***1^-resonances of W7 are observed, a very large shift for (dT)_7_, a moderate shift for (dC)_7_, and a very weak shift for (dA)_7_. This suggests that the two tryptophan residues take part in the recognition of the thymine–base. If the shifts of the tryptophan H^ε1^-resonances are mainly ring-current shifts by the bases, it indicates that thymine is optimally oriented relative to the tryptophan residues and that also the adenine ring has to come close to W29 but with an orientation different from the pyrimidine bases.

Under our conditions the binding site of a *Tm*Csp molecule for (rU)_7_ or (dT)_7_ may be only 3 nucleotides long. In consequence, the question arises whether this is true for selected heptapeptides with different nucleotide sequences. Csp from *T. maritima* is the only one which possesses a tryptophan residue (W29) instead of a highly conserved phenylalanine or tyrosine residue at the end of RNP2 [8]. During the 1D NMR titration experiments, the chemical shift of this residue (W29) changed to different extents according to the kind of oligonucleotide added. The two pyrimidine nucleotides lead to a downfield shift of the H^ε1^-resonance of W29 with a larger shift for (dT)_7_. However, the high-field shift induced (dA)_7,_ is much larger and may indicate that in *Tm*Csp this tryptophan favors an interaction with an adenine residue.

### *Tm*Csp as an Important Sensor Switching to the Cold Shock Response

When discussing the *Tm*Csp-oligonucleotide interaction, it is important to have in mind that Csp itself is part of a temperature sensing system or even may be the main temperature sensor for switching on and off the cold shock response. Under cold shock conditions, cold shock proteins have two different cellular functions, namely (1) the unspecific inhibition of the expression of the majority of proteins at high Csp concentrations and (2) the specific activation of cold chock related proteins (for a review see e. g. [2]). At physiological growing conditions, these regulatory functions should be switched off.

At the millimolar concentrations of Csps present at cold shock conditions transcription as well as translation is downregulated completely as studies in cell free systems show. For *Tm*Csp 50% inhibition of the two processes is obtained at a concentration of 140 μM. Inhibition can completely be abolished by addition of the hepta-2′-desoxy-thymidylate (dT)_7_ that competitively binds to *Tm*Csp with high affinity [20]. This is in line with our binding data since all studied purine and pyrimidine heptanucleotides bind with sufficient micromolar affinity to single stranded RNA and DNA under cold shock conditions (Table 1). Especially, Csp seems to be able to form larger, probably more stable clusters on the nucleotides inhibiting transcription as well as translation. This is in agreement with our study that shows that at cold shock temperatures *Tm*Csp forms dimers with heptanucleotides. It is most likely that longer nucleotides would also lead to larger nucleoprotein complexes.

The second mechanism, initializing and propagating expression of heat shock related proteins would require recognition of specific nucleotide sequences with high affinity. For this aim the recognition of a sequence motif larger than three nucleotides would be required when only one Csp molecule would bind. However, the problem could be solved even with binding of a short sequence of three nucleotides recognized by a single Csp-molecule when more than one Csp would bind cooperatively to a longer nucleotide sequence as observed experimentally in this study.

Generally, a preference for pyrimidine based oligonucleotides was detected for *Tm*Csp as it had been earlier described for *Bs*Csp [5, 6]. This specificity is rather small at optimal growing conditions where also the affinity of Csp for all nucleotides studied here is quite low (Table 1). However, at cold shock conditions (303 K) the protein showed a nanomolar affinity for thymidylate or uridylate heptamers but binds with three orders of magnitude lower affinities to purine nucleotides and to the pyridine heptamer (dC)_7_. Under cold shock conditions for the affinity to the ssDNA or RNA templates (dT)_7_ and (rU)_7_ no major differences were observed in line with the dual function of Csp in transcription and translation.

We obtained most of our information concerning the oligonucleotide-Csp interaction at cold shock conditions. The gel shift assays were performed at room temperature with nucleotide concentrations in the nanomolar range (0.5 μM) and maximum Csp concentrations of 100 μM ((dA)_7_, (dC)_7_, (dG)_7_) or 1.5 μM (((dT)_7_, (rU)_7_). No clear binding activity was observed with any of the purine based substrates, while (dT)_7_ and (rU)_7_ show strong binding. The NMR titrations were performed at a Csp-concentration of 1 mM at 303 K. Because of this high concentration, they are not suited for a quantitative determination of dissociation constants but for the determination of the stoichiometry of binding (see above). Quantitative binding constants were obtained by fluorescence quenching (Table 1). At 303 K and Csp concentrations between 5 nM and 1 μM, the dissociation constants of (dT)_7_ and (rU)_7_ are very small with 4 and 10.8 nM, respectively. For (dA)_7_, (dC)_7_, and (dG)_7_ they are approximately 3 orders of magnitude higher with 5.0, 2.8, and 3.4 μM, respectively. As a consequence a preferential interaction of Csp with nucleotides containing uracil or thymine is to be expected, although at the typical total concentrations of Csp of 100 μM during cold shock [2] also unspecific interactions should occur. At an almost physiological growing temperature for *T. maritima* of 343 K, the affinity of Csp to the model single stranded is significantly lower. It is very similar for all nucleotides studied here (Table 1) that is the specific recognition of nucleotides is decreased much. Together with the reduced Csp concentration at physiological temperatures, it means that in the cellular environment most of the nucleotide interactions are switched off as observed.

The temperature switch can now be understood from the structural perspective since at high non-cold shock temperatures the interaction with residues interacting specifically with uracil or thymine bases at low temperatures have to be perturbed. Since NMR structures of *Tm*Csp at 303 K [8] and 343 K [36] are available, we have a structural basis for estimating differences of the cold shock and the physiological state with respect to nucleotide binding. From the chemical shift perturbation β-strands 1 (W7, D9) and 2 (G13, Y14, F16) and the loop region in between the two strands (S10, K11, K12) appear to be involved in the nucleotide interaction. At cold shock temperatures, the H^ε1^-resonance of W7 shows very large upfield shift after binding of (rU)_7_ or (dT)_7_ that is not observed for the other oligonucleotides studied here. This suggests that a specific interaction of these two bases with the tryptophan ring exists causing an upfield ring current. At physiological high temperature this region is destabilized, especially a partial melting of β-strand 1 is observed including amino acids K6 and W7 that characteristically change their orientation in the structure [36]. A second characteristic temperature induced large conformational change was observed for the large loop (E52 to P57) between β-strand 4 and β-strand 5. At 303 K and 343 K, it adopts a well-defined orientation that differs significantly, at 328 K an equilibrium between these orientations is observed. Again, significant chemical shift perturbation after binding are observed here (G53, K55).

Directly after switching to low temperature, the Csp concentration is still rather small. Here, even higher affinity and specificity may be required. This suggests that more elaborated nucleic acid sequences may be recognized. It is likely that they contain at least a short sequence of rU or dT (probably three units/Csp according to our data). If a longer sequence would be required, the presence of at least one different base in a well-positioned place within the thymidylate-rich nucleic acid sequence would make sense. A cytidine is not a likely candidate because of the relative weak response in the NMR-spectra of the Csp (dC)_7_ complex (Fig. 2). One the other hand, the (dA)_7_ has a very strong shift effect on the H^ε1^ resonance of W29 suggesting a stacking interaction of its base with the tryptophan side chain. One can speculate what the recognition site of *Tm*Csp may look like. When we assume that (1) the binding of two *Tm*Csps is preferential, that (2) RNP1 also is a physiological interaction site requiring the pyrimidine nucleotides dT or rU in the *Tm*Csp-complex, and that (3) a direct interaction with the purine nucleotide (dA)_7_ and W29 at the end of RNP2 is proposed from the NMR-data, then a palindromic sequence like AUUUUUUA and UUUAUUU (or ATTTTTTA and TTTATTT in case of DNA binding) would satisfy these conditions.

Since we did not test the oligonucleotide (dG)_7_ by NMR, we cannot exclude that here the guanine base is even more specific. These findings support the notion that W29 plays a major role in the binding process. It is conceivable that in the case of (dT)_7_ or (rU)_7_ some steric hindrance occurs because of the non-optimal intercalation of W29 into these oligonucleotides which blocks the access of the RNP2 site to the DNA or RNA.

Future experiments will include an *in vitro* selection approach (SELEX) [27] to determine the optimal nucleotide binding sequence for *Tm*Csp. Additionally, a point mutation experiment will be performed, in which W29 will be mutated to a phenylalanine or tyrosine residue, which are highly conserved in the sequences of cold shock proteins of most species. In this way we will be able to distinguish whether the dimerization of *Tm*Csp was caused by the use of non-optimal templates, or whether such a short nucleotide sequence represents its true binding site and therefore a new binding mode. The excellent binding affinity found for the pyrimidine templates would argue for the latter possibility.

## Conclusion

At 343 K, close to the physiological temperatures for optimal growth of *T*. *maritima* [2] of 353 K, the affinities of all DNA and RNA homopolymers studied is relatively low, indicating that specific cold shock response by *Tm*Csp is switched off. The obtained affinities towards the homopolymers is close to 3.4 μM (at 353 K) estimated earlier in an *in-vitro* transcription-translation system for arbitrary genes [20]. Under cold shock conditions the affinities of (dT)_7_ and (rU)_7_ increase by three orders of magnitude, whereas the other oligonucleotides do not change their affinity much. This would perfectly fit to a picture where as specific cold shock response Csp would recognize nucleotide sequences containing poly-U or poly-T, but where the affinity of Csp after its millimolar concentration has been reached is sufficient to inhibit general protein expression. Here also multimerization of the bound Csp molecules could strengthen the inhibitory effect. The specific recognition sequences required for activation of the expression of cold shock induced proteins are not known yet but probably are rich in rU or dT. From the side of Csp, the unspecific inhibitory effect on protein transcription and translation does not require specific, temperature dependent conformational changes, since even at physiological temperatures its affinity to a variety of sequences is sufficient. However, the specific increase of affinity for poly-U or poly-T sequences under cold shock conditions requires specific conformational changes in the protein that switch on the cold shock response by a strongly increased affinity to the specific recognition sequences, analogous to the dramatic increase of the affinity for (rU)_7_ or (dT)_7_ by three orders of magnitude we observed here.

## Materials and Methods

### Protein Purification

For the expression of unlabeled protein, *E. coli* Rosetta (DE3) pLysS cells transformed with the plasmid coding for *Tm*Csp were grown in Luria Bertani medium in the presence of 50 µg/ml carbenicillin and 68 µg/ml chloramphenicol to an OD_600_ of 1, or, for the expression of ^15^N isotopically enriched protein, in New Minimal Medium [28] containing 1 g/ L ^15^NH_4_Cl and 2 g/ L glucose at 37°C to an OD_600_ of 0.8, respectively. Expression was induced by adding 1 mM isopropyl β-D-thiogalactopyranoside, and bacterial growth was continued for 3 h. Purification of the cold shock protein was performed as described [29]: to remove the bulk of the *E. coli* proteins without significant co-precipitation of *Tm*Csp, the cell extract was diluted fourfold with buffer and then heated to 70°C for 20 minutes. Pure *Tm*Csp was obtained by hydrophobic interaction chromatography at pH 8 and size exclusion chromatography. Its concentration was determined photometrically using an extinction coefficient ε_280_ of 12,660 M^−1^ cm^−1^.

### Oligonucleotides

All unlabeled and 5′-fluorescein-labeled ssDNA and RNA oligonucleotides were purchased in HPSF-quality (Highly Purified Salt Free) from MWG Biotech (Ebersberg, Germany) except for the unlabeled RNA, which was purchased from BioSpring (Frankfurt/Main, Germany). The concentrations of the unlabeled oligonucleotides were calculated from their absorbance at 260 nm. The extinction coefficients *ε*_*260*_ used for the individual oligomers were 15,400 M^−1^cm^−1^ for (dA)_7,_ 7300 M^−1^ cm^−1^ for (dC)_7_, 11,700 M^−1^ cm^−1^ for (dG)_7_, and 8400 M^−1^ cm^−1^ for (dT)_7_ and (rU)_7_ [30].

### NMR Spectroscopy

NMR samples of unlabeled and uniformly ^15^N-labeled *Tm*Csp contained 0.5 mM and 1 mM protein, respectively, in 50 mM NaH_2_PO_4_ (pH 6.5), 20 mM NaCl, 0.2 mM EDTA (sodium salt), 0.1 mM DSS in ^1^H_2_O/^2^H_2_O (92%/8%), and oligonucleotides in varying concentrations. The NMR experiments were carried out on a Bruker DRX-600 spectrometer (^1^H resonance frequency 600 MHz) at 303 K. ^1^H–^15^N heteronuclear single-quantum coherence (HSQC) spectra [31] were recorded with an echo/anti-echo-gradient selection in ω_1_ [32], the digital resolution was 4.75 Hz per point for ^15^N and 5.86 Hz per point for ^1^H.

The proton chemical shifts were referenced to sodium-2,2-dimethylsilapentane-5-sulfonic acid (DSS) used as an internal reference. ^15^N chemical shifts were referenced indirectly to DSS using an X-value of 0.101329118 [33]. Spectral analysis and peak picking were performed using the program AUREMOL (available at http://www.auremol.de) [34].

### NMR Data Evaluation

For the identification of the interaction sites, amino acid and atom specific combined chemical shifts [37] were calculated using the tool implemented in the program AUREMOL (www. auremol.de). The Euclidian norm was used. Note that the implementation in AUREMOL in its version from November 2019 now contains weighting factors for all types of atoms in the 20 amino acids (not only the backbone atoms as described in [37]). In our case not only the backbone N and H shifts were used but we could also include the effects on the tryptophan N^ε1^ and H^ε1^, as well as the amino groups of the glutamine side chains. The combined chemical shift perturbation can be applied for fast and slow exchange as long as the chemical shifts in the absence and presence of the ligands under saturation conditions are known. If in the slow or intermediate exchange region the chemical shifts in the complex could not be identified, they were treated separately. A significant interaction has been assumed

The probability *P*_*AB*_ that a molecule *Tm*Csp is bound to an oligonucleotide is given by (Eq. 1), where *c*_*A*_ and *c*_*B*_ are the total concentrations of *Tm*Csp and the heptanucleotide in solution, *N* is the number of independent binding sites on the heptanucleotide and *K*_D_ is the dissociation constant. Equation 1 assumes that there are *N* independent and equal binding sites.

1 $$P_{AB} = \frac{1}{2c_{A}} \left(c_{A} + Nc_{B} + K_{D} - \sqrt{(c_{A} + Nc_{B} + K_{D} )^{2} - 4Nc_{A} c_{B} }\right)$$

With *K*_D_ in the nanomolar and *c*_*A*_ and *c*_*B*_ in the millimolar range, Eq. 1 can be simplified to Eq. 2, neglecting *K*_D_.

2 $$P_{AB} = \frac{1}{2c_{A}} (c_{A} + Nc_{B} - \left|c_{A} - Nc_{B}\right| )$$

For signals under fast chemical exchange regime, the chemical shift *δ* is given by (Eq. 3). *δ*_*A*_ and *δ*_*AB*_ are the chemical shifts of free and bound protein. The relative change of the chemical shifts is given by Eq. 4 with *Δδ* *=* *δ* *−* *δ*_*A*_ and *Δδ*^*end*^ *=* *δ*_*AB*_ *−* *δ*_*A*_.

3 $${\delta} = P_{AB}\updelta_{{{\rm A}{\rm B}}} + (1 - P_{AB} )\updelta_{A}$$

4 $$\frac{\Delta \delta}{{\Delta \delta}^{end}} = P_{AB}$$

### Electrophoretic Mobility Shift Assay

The binding reaction was performed by incubating 7.5 pmol of 5′-fluorescein-labeled ssDNA or RNA with different amounts of protein in NMR buffer (50 mM NaH_2_PO_4_, pH 6.5, 20 mM NaCl, and 0.2 mM EDTA) at room temperature for 1h (total volume 15 µl). Prior to gel electrophoresis, 5 µl of dye solution (20% glycerol, 0.034% bromphenol blue) were added to the samples. Native gel electrophoresis was performed at 4 °C in 1xTBE buffer (5xTBE buffer: 54 g Tris, 27.5 g boric acid, 20 ml EDTA 0.5 M) through a 20% mini-polyacrylamide gel at 80 V until the dye had reached the bottom of the gel (ca. 7 h). The fluorescent oligonucleotides were detected by exposing the gels to UV light (302 nm). Then the gels were silver-stained to localize the position of the protein.

### Fluorescence Spectroscopy

The dissociation constants of *Tm*Csp complexed with various nucleotide heptamers (Table 1) were determined by quenching of the intrinsic tryptophan fluorescence of the nucleoprotein complex as described by Zeeb and Balbach [26] assuming a stoichiometry of two protein molecules bound to one oligonucleotide. Titration experiments at 303 K were carried out with protein concentrations of 5 nM ((dT)_7_), 25 nm ((rU)_7_) or 1 µM ((dC)_7_, (dA)_7_, (dG)_7_). For the determination of the *K*_*D*_ values at 343 K *Tm*Csp concentrations of 1 µM ((dT)_7_, (rU)_7_) or 5 µM ((dC)_7_, (dA)_7_, (dG)_7_) were used. The successive addition of oligonucleotides was performed until the protein was fully saturated with the complex partner and no further quenching of the Trp fluorescence was detectable.
